# Controlling the Gatekeeper: Therapeutic Targeting of Nuclear Transport

**DOI:** 10.3390/cells7110221

**Published:** 2018-11-21

**Authors:** Friederike K. Kosyna, Reinhard Depping

**Affiliations:** Institute of Physiology, Center for Structural and Cell Biology in Medicine, University of Lübeck, Ratzeburger Allee 160, D-23562 Lübeck, Germany; reinhard.depping@uni-luebeck.de

**Keywords:** nuclear transport, exportin, importin, karyopherin, chromosome region maintenance 1 (CRM1), cancer, drug, nuclear transport inhibitor

## Abstract

Nuclear transport receptors of the karyopherin superfamily of proteins transport macromolecules from one compartment to the other and are critical for both cell physiology and pathophysiology. The nuclear transport machinery is tightly regulated and essential to a number of key cellular processes since the spatiotemporally expression of many proteins and the nuclear transporters themselves is crucial for cellular activities. Dysregulation of the nuclear transport machinery results in localization shifts of specific cargo proteins and associates with the pathogenesis of disease states such as cancer, inflammation, viral illness and neurodegenerative diseases. Therefore, inhibition of the nuclear transport system has future potential for therapeutic intervention and could contribute to the elucidation of disease mechanisms. In this review, we recapitulate clue findings in the pathophysiological significance of nuclear transport processes and describe the development of nuclear transport inhibitors. Finally, clinical implications and results of the first clinical trials are discussed for the most promising nuclear transport inhibitors.

## 1. Introduction

The cytoplasm and the nucleoplasm are separated by the nuclear envelope in eukaryotic cells. Spatially segregation of essential cellular processes requires tight control of large molecule exchange such as RNAs, proteins, or ribonucleoprotein particles through this double membrane. The gatekeepers of these processes are nuclear pore complexes (NPC) which are large membrane-spanning protein complexes embedded in the nuclear envelope and consisting of multiple copies of approximately 30 different proteins called nucleoporins (Nups). They allow the passive passage of ions and molecules across the nuclear envelope, while building a barrier to free diffusion for molecules larger than a Stokes radius of ~2.5 nm, corresponding to a protein mass of approximately 35–40 kDa. The transfer of macromolecules such as proteins through the NPCs is strictly controlled by processes that involve a number of nuclear transport receptors (NTRs) called karyopherins or importins/exportins. In recent years, a wide variety of non-conventional nucleocytoplasmic transport processes have become increasingly apparent including karyopherin-dependent and –independent pathways [1]. However, this review seeks to discuss karyopherin-dependent processes, their physiological and pathophysiological roles and especially the current understanding of nuclear transport inhibition.

The nuclear transport machinery is essential to a number of key cellular processes [2,3]. Localization shifts of specific cargo proteins can lead to the dysregulation of individual pathways, as well as physiological and pathological alterations. Therefore, inhibition of the nuclear transport system has potential for therapeutic intervention and could contribute to the elucidation of disease mechanisms in the future. Herein, we summarize and discuss specific and general inhibitors of protein nuclear transport receptors and their clinical implications.

## 2. Karyopherins: Key Molecules in Nuclear Transport

Karyopherins transfer the majority of proteins through the NPC into the nucleus. The karyopherin superfamily consists of the importin β (karyopherin β) and the importin α (karyopherin α) subfamily of soluble nuclear transport receptors which possess different structural and functional features. All members of the superfamily contain tandem huntingtin, elongation factor 3, protein phosphatase 2A and mechanistic target of rapamycin (HEAT) repeats in their secondary protein structure which contain ~ 40–45 amino acids and form two antiparallel α-helices linked by a loop [4].

The human genome encodes at least 20 importin β isoforms. Based on the direction in which karyopherins transport their cargo proteins, they are termed importins or exportins. Ten importin β karyopherins are involved in nuclear import (importin β1/KPNB1, transportin 1/TNPO1, transportin 2/TNPO2, importin 4/IPO4, importin 5/IPO5, importin7/IPO7, importin 8/IPO8, importin 9/IPO9, importin 11/IPO11 and importin 12/IPO12), six in nuclear export (chromosome region maintenance 1 (CRM1/XPO1), cellular apoptosis susceptibility (CAS/CSE1L), exportin 5/XPO5, exportin 6/XPO6, exportin t/XPOT and RanBP17/RANBP17) and three importin βs (exportin 4/XPO4, exportin 7/XPO7 and importin 13/IPO13) mediate bidirectional transport. Until now, one importin β isoform (RanBP6/RANBP6) remains uncharacterized [1,5]. Some importin βs recognize their cargo proteins directly via specific interactions with signal sequences, namely nuclear localization signals (NLS) or nuclear export signals (NES). The import receptor transportin 1, for example, recognizes a proline-tyrosine-rich NLS in the primary amino acid sequence of the cargo proteins [6], whereas the export receptor CRM1 binds to a leucine-rich NES [7]. Other cargo proteins require additional adaptor proteins that link them to the main karyopherin. For example, asp-glu-ala-asp (DEAD)-box helicase 6 binds to eukaryotic translation initiation factor 4E (EIF4E) nuclear import factor 1/transporter for nuclear export via the CRM1-dependent pathway [8]. The heterodimer consisting of importin β and importin 7 transports histone H1 into the nucleus. Thereby, importin 7 resembles an import adapter, while importin β represents the main import receptor [9]. However, the best studied adaptors for nucleocytoplasmic transport belong to the importin α family of proteins and mediate the classical nuclear import pathway which is discussed in detail below.

In the human genome seven importin α isoforms are encoded, which are named importin α1 to importin α7 (KPNA1 to KPNA7). Importin α possesses the indispensable role of ferrying proteins from the cytoplasm to the nucleus in combination with a transport carrier [10]. Interestingly, only importin β1 uses importin α adaptor proteins [11]. The adaptor protein importin α dimerizes with importin β1 and binds the cargo protein via a classical NLS (cNLS) which is rich in lysine and arginine and exemplified by the monopartite SV40 large T-antigen cNLS [12]. Building up the trimeric complex is mandatory for the translocation process [13]. Subsequently, the transport of the trimeric cNLS/importin α/importin β protein complex into the nucleus is facilitated by direct interaction between importin β and the NPC [14]. For a growing number of cellular cargo proteins it was shown that they rely on the classical nuclear import pathway for transport into the nucleus including NF-κB (p50/p65) [15] or the hypoxia-inducible factors (HIF) 1 and 2 [16]. Many independent variables like the expression levels of the karyopherins, posttranslational modifications and masking or unmasking of the transport signals by interacting proteins are modulators of nucleocytoplasmic transport [17,18]. Important aspects of the modulation of nuclear protein transport are post-translational modifications. One example is sumoylation of bovine papillomavirus E1, wherefore sumoylation at residue K514 is a prerequisite of its nuclear localization [19].

**Figure 1:**
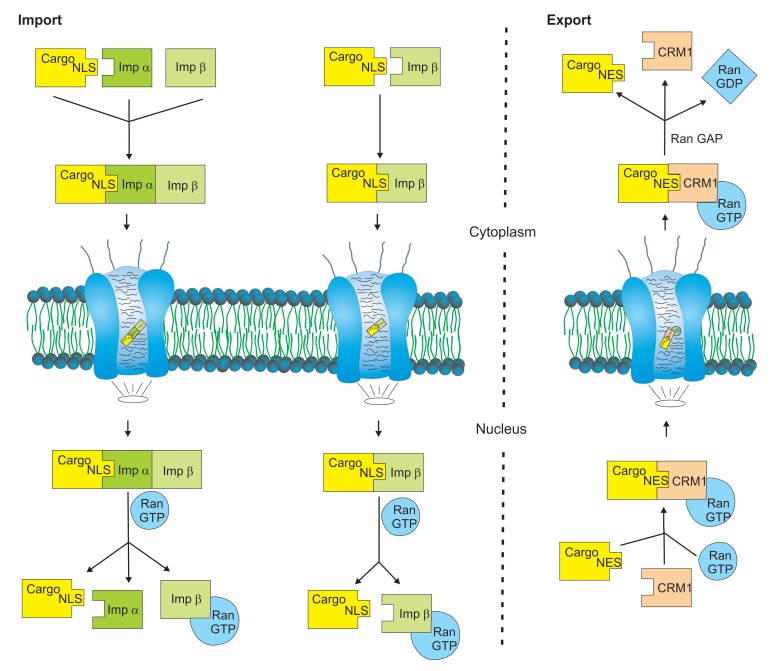
Simplified model of nuclear import and export processes. The transfer of cargo proteins (yellow) between nucleus and cytoplasm occurs through the nuclear pore complex and is mediated by members of the karyopherin superfamily. Nuclear import is mediated by importin β and importin α nuclear transport receptors (green). Some importin βs recognize their cargo proteins directly via a nuclear localization signal (NLS), while others require importin α adaptor proteins. Nuclear export mainly occurs via the transport receptor chromosome region maintenance 1 (CRM1) (light red) and depends on nuclear exclusion signals (NES) in the primary amino acid sequence of the cargo proteins. Nuclear import and export cycles depend on the Ras-related small GTPase Ran (light blue) and the hydrolysis of guanosine triphosphate (GTP).

The Ras-related small GTPase Ran regulates the conformation of importin β and determines molecular interactions between the nuclear transport receptor and its cargo protein [20]. Importin β is recognized by guanosine triphosphate-bound Ran (RanGTP), but guanosine diphosphate- bound Ran (GDP) dissociates from importin β. Ran is predominantly bound to GTP in the nucleus and to GDP in the cytoplasm indicating that the Ran concentration gradient makes a major contribution to vectorial transport directionality. While some importin βs (importins) interact with their cargo proteins in the absence of RanGTP, others (exportins) bind to their cargo proteins in the RanGTP bound state. After protein transport into the nucleus, RanGTP tightly binds to the importin β transport receptor resulting in structural alterations and dissociation of the trimeric NLS/importin α/importin β protein complex. Conversely, exportins have to associate with RanGTP to enable stable binding of their cargo proteins. Following nuclear export through the NPC, RanGTP hydrolysis to RanGDP is catalyzed by RanGAP leading to dissociation of the trimeric NES/CRM1/RanGDP protein complex [21,22]. Figure 1 provides a summary of nuclear import and export processes.

## 3. Functional Diversities of Nuclear Transport Receptors

The large number of nucleocytoplasmic transport receptors displays the diverse functions of karyopherins. Karyopherin-dependent transport is involved in various key processes such as gene expression, signal transduction, immune response, oxygen sensing, development, circadian rhythm and spindle assembly [16,23,24,25]. This functional diversity requires interaction with a large number of different proteins via distinct domains of the nuclear transport receptors. Remarkably, one karyopherin can import and/or export a high number of different cargo proteins using different binding sites and distinct conformational stages. In yeast and mice, expression analysis of karyopherins revealed that their transcription is regulated probably due to varying requirements for transport both during cell cycle and development [26].

In addition to the significant impact of karyopherins in nucleocytoplasmic transport, a growing number of studies reveal diverse non-transport functions of importin αs and importin βs. Interestingly, import receptors have been proposed to function as cytoplasmic chaperones. In the cytoplasm, they cover extremely basic domains of some proteins, thereby preventing their aggregation [27]. Some nuclear proteins use transport along microtubules as a nuclear import route. In this context, importin β may function as adaptor linking cargo proteins such as the small protein PTHrP to the microtubules [28]. Moreover, karyopherins have been associated with gene-specific regulation processes. It could be demonstrated that hydrogen peroxide exposure causes nuclear retention of importin α2. This results in binding of importin α2 to the promoter region of Serine/threonine kinase 35, thereby coordinating cell fate through changes in gene expression [29]. To date, the best-characterized non-transport function of the karyopherins is their involvement in the global regulation of mitosis. Here, karyopherins are involved in different aspects of spindle assembly as well as the regulation of nuclear envelope and pore assembly [30].

## 4. Pathophysiological Relevance of Nucleocytoplasmic Transport

The activity of a given protein depends to large extends on its correct spatial arrangement at the correct time. Therefore, it is not surprising that the regulation of the expression, the localization and the biological activity of the karyopherins has to be tightly regulated. However, various increments of the control processes entail multiple contingencies of defectiveness. In this line, it is clear that dysregulation of the nucleocytoplasmic transport of specific cargo proteins is associated with the pathogenesis of disease states such as cancer, inflammation, viral illness and neurodegenerative maladies. The many possible junctions at which dysregulation may occur include upstream cell signaling events, modifications of the cargo proteins or the karyopherins and the ability of the cargo protein to recognize and bind its karyopherin.

Impaired regulation of nuclear import or export of drug targets, tumor suppressors or cell cycle inhibitors contributes to cancer development. In breast cancer cells, for example, nuclear import of p53 is inhibited by the expression of an importin α mutant with a truncation in the NLS-binding domain resulting in cytoplasmic retention of p53, inactivation of its tumor suppressor functions and tumorigenesis [13]. Moreover, the transcription factor Wt1 accumulates in the cytoplasm of malignant cells leading to the hypothesis that a disparate subcellular localization of Wt1 in normal and cancer cells contributes to its dual functions as tumor suppressor and oncogene [31]. In chronic myelogenous leukemia the chimeric BCR-ABL oncoprotein activates mitogenic and anti-apoptotic pathways in the cytoplasm, while nuclear entrapment is a promising strategy to attack bone marrow cells that express BCR-ABL [32]. Mislocalization of cancer-associated cargo proteins has also been implicated in gastric cancer progression by nuclear accumulation of the DNA repair protein NBS1 and in Hodgkin’s lymphoma and childhood acute lymphoblastic leukemia due to nuclear accumulation of the transcriptional activator nuclear factor-κB (NF-κB) [33,34].

In recent times, a prognostic role of nuclear transport proteins was established. There is strong evidence that the expression and localization of nuclear transporters associates with clinical outcome in cancer. CRM1, for example, has been identified as a suitable marker for poor prognosis in gastric, ovarian, pancreatic and brain cancer as well as in acute myeloid leukemia and osteosarcoma [35,36,37,38,39,40]. Moreover, importin β1 has been identified as marker for poor prognosis in gastric cancer [41], while the nuclear expression of the export receptor CAS associates with lower overall survival and highly aggressive tumors in bladder and ovarian cancer, respectively [42,43].

In addition, karyopherins play a fundamental role in the upregulation of inflammatory gene networks and the innate immune response. For example, in human studies of sepsis the persistent nuclear localization of NF-κB in blood monocytes showed strong correlation with an ultimately fatal outcome [44]. Nucleocytoplasmic transport is also critical to productive virus infection and the life cycle of many viruses. In the case of human immunodeficiency virus (HIV) viral infection depends on the nuclear import of the HIV-1 integrase protein via the classical nuclear import pathway [45,46]. Moreover, Nuovo et al., suggested a prognostic role of karyopherins for acute viral infection [47]. They detected an increased expression of importin β and exportin 5 in cells infected with a panel of different viruses by immunohistochemistry. Very recently, it could be demonstrated that importin α3 is a key player in the pathogenesis of spinocerebellar ataxia type 3 providing a direct link of nucleocytoplasmic transport to the pathogenesis of neurodegenerative diseases [48]. Moreover, defects in nucleocytoplasmatic transport of HIF have been associated with the pathogenesis of motor neuron diseases like amyotrophic lateral sclerosis (ALS). Cytoplasmatic HIF-1α levels are increased in ALS suggesting impaired nuclear transport of HIF-1α in ALS. Accordingly, Grima et al. showed that Huntington’s disease (HD) is caused by aggregation of several Nups and defects in cytoplasmic transport [49]. Interestingly, nuclear import seems to be especially deficient in HD. The high number of pathophysiological processes described herein and in many other studies highlights the fact that the nuclear transport machinery displays a promising therapeutic target [50,51,52,53].

## 5. Targeting Nucleocytoplasmic Transport

The high pathophysiological relevance of the karyopherins highlights their potential as therapeutic targets to attack various diseases. The lack of compounds which inhibit nuclear transport of specific cargo substrates hampers the development of therapeutic strategies. However, it could be shown that cancer cells are more sensitive to inhibition of the nuclear transport machinery than non-cancer cells due to their increased proliferative and metabolic demands [54,55]. Therefore specific protein nuclear transport inhibitors alone or in combination with other medical treatments such as chemotherapeutic or antiviral agents might be a promising strategy to target major life-threatening diseases.

### 5.1. Nuclear Export Inhibition

So far, the development of nuclear export inhibitors was more successful than that of nuclear import receptors. Since numerous studies in this field have been published in the last year, Table 1 summarizes selected nuclear export inhibitors. In addition, previous review articles show detailed information about nuclear export inhibtors in anticancer or antiviral therapy [56,57].

CRM1 is the major receptor for the export of proteins out of the nucleus and has been the only karyopherin targeted for nuclear export inhibition for many years. Pioneering work in the search for nuclear export inhibitors has been achieved by Nishi et al. among others by the characterization of Leptomycin B (LMB, Elactocin), a specific CRM1 inhibitor [76]. Afterwards, in fall 1997, four groups expanded the potential of LMB by describing CRM1 and its role as nuclear export receptor [77,78,79,80]. LMB covalently binds to cysteine 528 in the central conserved binding region of CRM1, thereby directly blocking its interaction with the NES of a cargo protein in an irreversible manner [76,81,82]. LMB has been widely used as a cell biological tool leading to the discovery of hundreds of broadly functioning nuclear export cargos such as p53 and breast cancer 1 (BRCA1) [83]. LMB was tested in phase I clinical trials to treat advanced refractory cancer (Table 2). However, due to significant systemic toxicity and limited efficiency in both animals and humans, clinical trials with LMB were not continued [84].

Subsequently, a series of LMB analogs with improved therapeutic windows have been developed. Semisynthetic derivates of LMB such as Anguinomycin C and D were originally isolated as a natural product and also chemically synthesized. Interestingly, they inhibit nucleocytoplasmic transport in picomolar concentrations and cause apoptosis in tumor cell lines, while inducing growth arrest against normal cells [59,92]. Ratjadones, another class of natural LMB derivates, were isolated from the myxobacterium Sorangium cellulosum. CRM1 dependent nuclear export is inhibited by Ratjadones employing the same molecular mechanism as LMB. Cell-cycle studies in bacteria, in yeast, and in several human cancer cell lines revealed that Ratjadones inhibit cell growth and proliferation by arresting the cells in G1 phase [60]. Moreover, it could be demonstrated that Ratjadone A exhibits strong anti-viral activity in HIV infection studies. However, its therapeutic potential is limited by low selectivity and toxic side effects [93]. Another natural occurring lactone interfering with nuclear export is Goniothalamin which is extracted from root and bark of the genus Goniothalamus (Annonaceae). This compound triggers antiproliferative activity and caspase-induced apoptosis in cancer cells [61]. The molecular mechanism of action of Goniothalamin is comparable to that of LMB resulting in a strong inhibition of CRM1-dependent nuclear export [94]. By the synthesis of LMB derivates a new class of nuclear export inhibitors was established of which KOS-2464 was identified as the most effective LMB analog. In mouse models, KOS-2464 resulted in rapid and prolonged block of CRM1-mediated nuclear export which was achieved with 16-fold higher tolerability of the compound. KOS-2464 was also found to induce apoptosis in cancer cells, tumor regression and/or tumor growth inhibition [62]. However, to the best of our knowledge, all the derivates and analogues of LMB have not been studied in vivo in the clinical setting so far.

Another synthetic CRM1 inhibitor is CBS9106 (SL-801). This compound does not block the interaction between CRM1 and the NES of a cargo protein, but inhibits nuclear export through CRM1 degradation which is mediated by cullin ring ligase activity and the neddylation pathway [63]. The clinical potential of this candidate is currently being investigated in a phase I clinical trial in adult patients with locally advanced, unresectable or metastatic solid tumors (Table 2). The HIV Rev protein promotes the export of unspliced and partially spliced mRNA in a CRM1-dependent manner. The low molecular weight compound PKF050-638 could be identified as specific inhibitor of Rev-function interfering with Rev/CRM1 complex formation. The molecular mechanism by which PKF050-638 disrupts CRM1-NES interaction is similar to that of LMB [64]. However, this synthetic compound has only been used for studying CRM1-mediated export pathways in vitro. Mislocalization to the cytoplasm of Forkhead family of transcription factors (FOXO) is involved in tumorigenesis [95,96]. Therefore, Kau et al., performed a chemical genetic screen in order to identify inhibitors of CRM1-dependent nuclear export of FOXO [65]. Eleven compounds were found to inhibit nuclear export of FOXO targeting cystein 528 of CRM1. The two plant-based small-molecule CRM1 inhibitors Valtrate and acetoxychavicol acetate were identified as promising scaffolds for new anti-viral agents [66,67]. As LMB, both compounds inhibit nuclear export of the HIV Rev protein and influenza viral ribonucleoprotein by binding to the central conserved region of CRM1. More potent analogs of these synthesized compounds are still under investigation [97].

The further development of nuclear export inhibitors by structure-based drug design led to small molecule inhibitors of CRM1, also known as selective inhibitors of nuclear export (SINE) compounds [68,98,99]. SINE compounds, including KPT-185, KPT-251, KPT-276, KPT-330 (Selinexor) and KPT-335 (Verdinexor), are orally bioavailable and highly selective [68,69,70]. As does LMB, these compounds interfere with cysteine 528 in the NES-binding pocking of CRM1. However, binding to other proteins like cysteine proteases could not be detected which has been discussed to be the reason for poor tolerance of LMB [100]. Although preliminary studies investigating SINE compounds have concentrated on their use as anti-cancer agents, recent studies suggest Verdinexor as a novel broad-spectrum antiviral against various influenza strains and respiratory syncytial virus [101,102]. The anti-viral activity of Verdinexor was underlined by Widman et al. showing that Verdinexor efficiently inhibits infection by a panel of different viruses (Epstein-Barr virus, human cytomegalovirus, Kaposi’s sarcoma virus, adenoviruses, BK virus, John Cunningham virus, and human papillomavirus) in antiviral screens [103]. As many viruses use the host nucleus as part of their viral life cycle, SINE compounds will be further analyzed for their therapeutic potential as antivirals. Moreover, SINE compounds were suggested to be suitable candidates for the development of therapeutic strategies in neurodegenerative diseases. In preclinical models of inflammatory demyelination and another model of axonal damage CRM1 inhibition resulted in attenuation of disease progression [104]. In this line, CRM1 inhibition showed neuroprotective effects in ALS models related to hexanucleotide repeat expansion in C9orf72 [105]. KPT-8602 (Eltanexor) is a second-generation SINE compound with markedly reduced penetration across the blood−brain barrier and a substantially better tolerability profile compared to other SINE compounds [71,72,73]. SINE compounds, generally, cause nuclear accumulation of tumor suppressors and cell cycle inhibitors and sensitize resistant cancer cells to other drugs. The anti-cancer activity of SINE compounds in combination with standard therapies achieves high remission rates and has been highlighted by different studies [106,107,108,109]. For example, in multiple triple-negative breast cancer (TNBC) models, antitumor efficacy of Selinexor increased in combination with paclitaxel or eribulin [110]. Accordingly, Selinexor inhibited multiple myeloma tumor growth and increased survival in mice in combination with the proteasome-inhibitors bortezomib and carfilzomib [111]. Some of the SINE compounds are currently tested in phase I/II/III clinical trials to treat solid organ malignancies as a single agent or in combination with standard therapies. A selection of these clinical trials is presented in Table 2. Detalied information is available from other excellent reviews covering data available from a high number of clinical trials [112,113,114,115,116]. To sum up, the results of these trials are encouraging, as they have shown that SINE compounds in general, and Selinexor in particular, are active in heavily pretreated cancer conditions [72,86].

Very recently, two natural agents known for their anti-inflammatory, anti-carcinogenic and anti-oxidant properties were identified as inhibitors of the CRM1-dependent nuclear export. Curcumin is the main constituent of turmeric which is an old Indian spice. By elucidation of the molecular mechanisms underlying its diverse biological effects, CRM1 was identified as a specific target of curcumin. Mass spectrometric analysis as well as in vivo experiments revealed that Curcumin specifically disrupts the interaction between the conserved cysteine 528 of CRM1 and the NES of the cargo protein [74]. Interestingly, also the caffeic acid phenethyl ester (CAPE) which is the bioactive constituent of propolis from honeybee hives targets the NES-binding pocket of CRM1 at cysteine 528 [75]. These findings are of high interest for further clinical investigation of Curcumin and CAPE and the further characterization of plant-based protein nuclear export inhibitors.

### 5.2. Nuclear Import Inhibition

To date, the development of protein nuclear import inhibitors lags behind that of nuclear export inhibitors. Progress has been made in recent years targeting the activity of several nuclear import receptors (Table 3). Although nuclear import inhibitors are widely used as biological tools to identify karyopherin cargo proteins, they have as yet not entered clinical trials.

In 1995, cell permeable peptides were devised by Lin et al. controlling agonist-induced nuclear translocation of transcription factor κB (NF-κB) in intact cells [117]. A hydrophobic region of the signal peptide sequence interacts with lipid bilayers and was used as a carrier to deliver a functional NLS into the cell. The functional cargo representing the nuclear localization sequence of the transcription factor NF-κB p50 subunit inhibited nuclear translocation of NF-κB/Rel complexes from the cytoplasm to the nucleus in a dose-dependent manner. Later on, the same peptide was used to inhibit nuclear translocation of other transcription factors containing a NLS such as nuclear factor of activated T-cells (NFAT), AP-1 and signal transducer and activator of transcription 1 [127]. Meanwhile this peptide is known as cSN50.1 and has been used in inflammation research for many years investigating the therapeutic potential of targeting nuclear transport in inflammatory diseases such as sepsis and atherosclerosis [128,129].

For the identification of small molecule inhibitors of the importin α/β pathway a new screening approach was developed by Ambrus et al. [118]. They adapted a permeabilized cell nuclear import assay to 96-well plate format and screened peptidomimetic libraries. By this, 60 independent compound mixtures were identified significantly inhibiting the importin α/β pathway. Rescreening some individual compounds of the mixtures such as 58H5-6 revealed that they potently inhibit importin α/β-mediated nuclear import in vitro. However, further chemical modification and optimization of 58H5-6 and other inhibitors is necessary since no inhibitory effects could be observed in vivo, possibly due to insufficient cell permeability [118]. In a similar way, Hintersteiner et al. performed a library scanning method based on confocal detection of fluorescently labeled proteins bound to bead-immobilized compounds in order to identify small molecule inhibitors of nuclear import [119]. Interestingly, karyostain 1A was identified as potent inhibitor of importin α/β-mediated nuclear import at low micromolar concentrations. The molecular interaction between importin β and the GTPase Ran is targeted by karyostatin 1A in vitro as well as in vivo. By a fluorescence resonance energy transfer (FRET)-based, high-throughput small molecule screen importazole (2,4-diaminoquinazoline) was identified as an inhibitor of the importin α/β-mediated nuclear import [120]. Importazole could be shown to target the interaction between RanGTP and importin-β. By further investigating the role of nuclear import inhibition throughout the cell cycle, Soderholm et al. demonstrated that disruption of the Ran/importin-β interaction affects cell cycle regulation by causing spindle misalignment [120]. In the last years, the value of importazole in the academic field became apparent by studies describing the identification of nuclear import receptors for several proteins [130,131]. The inhibitor of nuclear import-43 (INI-43, (3-(1*H*-benzimidazol-2-yl)-1-(3-dimethylaminopropyl)pyrrolo[5,4-*b*]quinoxalin-2-amine)) was shown to specifically interfere with the nuclear localization of importin β and its cargo proteins NFAT, NF-κB, AP-1 and nuclear transcription factor Y. Moreover, INI-43 inhibits the proliferation of cancer cells of different tissue origins by induction of the intrinsic apoptotic pathway [121]. The nuclear import inhibitor 2-aminothiazole derivative 1 is the latest described small molecule inhibitor of nuclear import. This inhibitor, like INI-43, specifically targets importin β and causes cell cycle arrest in the G2/M phase underlining its potential as anticancer agent [122]. On the basis of structure-based design in combination with biochemical analyses the nuclear import inhibitor M9M has been developed [123]. M9M is a peptide inhibitor which specifically targets transportin 1-dependent nuclear import and binds transportin 1 200-fold tighter than NLSs of different transportin 1 cargo proteins. Kosugi et al. developed a new additivity-based method for the design of peptide inhibitors specifically targeting importin α/β-mediated nuclear import [124]. Indeed, the two peptides Bimax1 and Bimax2 possess an extremely high affinity for binding of full-length versions of importin α, but not to importin β or other karyopherins. It was also suggested that bimax1 and bimax2 antagonize release of the importin α/β-cargo proteins to restrict importin α recycling and deplete a pool of free importin α. The bimax inhibitors possess a high potency and specificity to inhibit importin α/β-dependent transport. Despite the high potential of M9M and bimax1/2 for studying cellular signaling events involving nuclear import, the therapeutic applicability of these peptide inhibitors has not been investigated yet.

In order to establish the antiviral activity of protein nuclear import inhibitors, Wagstaff et al. applied a high-throughput screening assay based on amplified luminescent proximity homogeneous assay technology [125]. They detected specific interactions between the integrase molecule of HIV-1 and its nuclear import receptors importin α/β. Several compounds were identified to target the IN/importin α/β interaction in vitro as well as in living cells. Mifepristone and ivermectin were shown to be the most potent broad-spectrum inhibitors of importin α/β-mediated transport, targeting no other nuclear import pathways. These inhibitors were used to investigate intracellular localization of several viral proteins and viral reliance on host nuclear transport processes during infection [132]. Moreover, potent antiviral activity towards HIV-1, dengue virus (DENV) and Venezuelan equine encephalitis virus (VEEV) of mifepristone and ivermectin could be established [133,134]. Ivermectin protects against infection by the four serotypes of DENV and ivermectin as well as Mifepristone lower the level of VEEV titers and cytopathic effects associated with VEEV infection [134,135]. The potent antiviral activity of ivermectin could also be demonstrated by targeting host-virus interactions in Hendra virus infection [136]. Moreover, therapeutic applicability of ivermectin could be expanded by detecting anti-parasitic effects of ivermectin inhibiting intracellular dynamic structures of the parasite Plasmodium falciparum [137]. In our lab, for the first time, we studied the effect of a protein nuclear import inhibitor, in general, and ivermectin, in particular, on the hypoxia response pathway [138]. We could demonstrate that ivermectin inhibits the hypoxia response pathway on the molecular level underlining the physiological activity of nuclear import inhibition. In this line, it is well worth considering that the hypoxia response pathway plays a pivotal role in the cellular adaptation to reduced oxygen levels which is one of the main features of solid tumors. Last year, the fundamental role of protein nuclear trafficking in virus infection could be expanded by Wang et al. identifying the compound N-(4-hydroxy-phenyl) retinamide (4-HPR) as antiviral agent to combat zika virus (ZIKV) infection [126]. 4-HPR inhibits specific binding of ZIKV nonstructural protein 5 to the importin α/β heterodimer and blocks ZIKA infection. These findings emphasize that targeting nuclear import has great potential for the generation of clinical strategies in major diseases such as cancer or viral infection.

Plasmid-encoded nanobodies are a newly described class of protein nuclear transport inhibitors. Aksu et al. developed these camelid-derived single-domain antibodies in order to validate cargo proteins of the nuclear import and export receptor exportin 7 [6]. Anti-exportin 7 (Anti-Xpo7) nanobodies specifically inhibit exportin 7 function when transfected into cultured cells and allow validation of numerous tested cargo candidates. Until now these nanobodies have not been associated with the development of clinical strategies. However, they have been described as powerful tools for cargo discovery, cargo validation and the analysis of exportin and importin function in complex systems.

## 6. Conclusions

Functioning of many proteins requires the tight regulation of its intracellular localization. Therefore, nuclear transport is of fundamental importance for a variety of physiological and pathophysiological processes. The targeted mislocalization of proteins has been proposed to be a promising strategy in the development of therapeutics. As a result, the field of protein nuclear transport inhibitors is growing. So far, more than 20 nuclear transport inhibitors have been described of which only a very few were monitored in a clinical setting. Further research is required to further establish protein nuclear transport inhibitors as an innovative class of therapeutic and prophylactic intervention in different diseases.

## Figures and Tables

**Table 1:** Selected protein nuclear export inhibitors.

Compound	Synonym	NTR	Type of Compound	Reference
Leptomycin B (LMB)	Elactocin	CRM1	Antibiotic	[58]
Anguinomycins		CRM1	Antibiotic	[59]
Ratjadones	-	CRM1	Antibiotic	[60]
Goniothalamin	-	CRM1	Small molecule (natural)	[61]
KOS-2464	-	CRM1	Small molecule (synthetic)	[62]
CBS9106	SL-801	CRM1	Small molecule (synthetic)	[63]
PKF050-638	-	CRM1	Small molecule (synthetic)	[64]
FOXO inhibitors		CRM1	Small molecule (synthetic)	[65]
Valtrate	-	CRM1	Small molecule (natural)	[66]
Acetoxychavicol acetate	-	CRM1	Small molecule (natural)	[67]
SINE series	-	CRM1	Small molecule (synthetic)	[68]
KPT-330 (SINE)	Selinexor	CRM1	Small molecule (synthetic)	[69]
KPT-335 (SINE)	Verdinexor	CRM1	Small molecule (synthetic)	[70]
KPT-8602 (SINE)	Eltanexor	CRM1	Small molecule (synthetic)	[71,72,73]
Curcumin	-	CRM1	Small molecule (natural)	[74]
Caffeic acid phenethyl ester (CAPE)	-	CRM1	Small molecule (natural)	[75]

**Table 2:** Selected clinical trials with protein nuclear export inhibitors.

Compound	Reference, Phase	Subjects	Treatment	ORR
Leptomycin B (Elactocin, NSC364372D)	[84], phase I (discontinued)	n = 33 advanced refractory cancer	LMB	No partial or complete responses
CBS9106 (SL-801)	NCT02667873, phase I (recruiting)	n = 40–50 advanced solid tumors	CBS9106	-
Verdinexor (KPT-335)	[85], phase II	n = 58 dogs B-cell and T-cell lymphoma	Verdinexor	37% (71% dogs with T-cell lymphoma)
Verdinexor (KPT-335)	NCT02431364, phase I	n = 32 healthy adult participants	Verdinexor	No results available
Eltanexor (KPT-8602)	NCT02649790, phase I/II (recruiting)	n = 119 refractory cancer conditions	KPT-8602	-
Selinexor (KPT-330)	NCT02336815, [86], phase II	n = 79 refractory multiple myeloma	Selinexor Dexamethasone	21% (18% treatment discontinuation )
Selinexor (KPT-330)	NCT01607892, [87], phase I	n = 25 heavily pretreated multiple myeloma or Waldenstrom macroglobulinemia	Selinexor Dexamethasone	4% without Dexamethasone, 50% with Dexamethasone
Selinexor (KPT-330)	NCT01607892, [88], phase I	n = 95 acute myeloid leukemia	Selinexor	14% objective response, 31% ≥50% decrease in bone marrow blasts
Selinexor (KPT-330)	NCT01607892, [89], phase I	n = 70 refractory non-Hodgkin lymphoma	Selinexor	31% (4% complete, 18% partial)
Selinexor (KPT-330)	NCT02606461, phase III (recruiting)	n = 222 advanced unresectable liposarcoma	Selinexor	-
Selinexor (KPT-330)	NCT02343042, [90], phase I (recruiting)	n = 42 relapsed or refractory multiple myeloma	Selinexor Bortezomib Dexamethasone	63% (84% nonrefractory, 43% refractory)
Selinexor (KPT-330)	NCT02215161, [91], phase II	n = 14 refractory metastatic castration-resistant prostate cancer	Selinexor	64% prostate-specific antigen (PSA) decline, poor tolerability

**Table 3:** Selected protein nuclear import inhibitors.

Compound	Synonym	NTR	Type of Compound	Reference
cSN50.1	-	Imp α/β, Impβ	Peptide	[117]
58H5-6	-	Imp β	Small molecule (synthetic)	[118]
Karyostatin 1A	-	Imp β	Small molecule (synthetic)	[119]
Importazole	-	Imp α/β	Small molecule (synthetic)	[120]
Inhibitor of nuclear import-43 (INI-43)	-	Imp β	Small molecule (synthetic)	[121]
2-aminothiazole derivative 1	-	Imp β	Small molecule (synthetic)	[122]
M9M	-	Transportin	Peptide	[123]
Bimax 1/Bimax 2	-	Imp α	Peptide	[124]
Ivermectin	Stromectol	Imp α/β	Antibiotic	[125]
Mifepristone	Mifegyne	Imp α/β	Small molecule (synthetic)	[125]
N-(4-hydroxy-phenyl) retinamide (4-HPR)	-	Imp α/β	Small molecule (synthetic)	[126]
Anti-Xpo7 nanobodies	-	Exportin 7	Nanobody	[5]
